# Antifungal Activity of Solventlessly Synthesized Organomercurials

**DOI:** 10.1155/MBD.2002.283

**Published:** 2002

**Authors:** M. Kidwai, R. Venkataramanan, B. Dave

**Affiliations:** Department of Chemistry University of Delhi Delhi 110007 India

## Abstract

A series of new barbituryl/thiobarbituryl substituted organomercurial derivatives 3a-i have been synthesised from pyrimidine derivatives 1a-c and arylmercuric chloride 2a-c over K_2_CO_3_ under microwave irradiations (MWI). This solventless synthesis apart from eliminating organic solvent from workup step, also gave improved yield as compared to the conventional heating, with reaction time reduced from hours to minutes. The prepared compounds were tested against A. niger and A. flavous for their antifungal activity and were found to posses good activity.


### ANTIFUNGAL ACTIVITY OF SOLVENTLESSLY SYNTHESIZED ORGANOMERCURIALS

M. Kidwai*, R. Venkataramanan and B. Dave

Department of Chemistry, University of Delhi, Delhi-110007, India
<mkidwai@mantraonline.com>

### ABSTRACT

A series of new barbituryl/thiobarbituryl substituted organomercurial derivatives 3a-i have been synthesised from pyrimidine derivatives 1a-c and arylmercuric chloride 2a-c over $K_2CO_3$ under microwave irradiations (MWI). This solventless synthesis apart from eliminating organic solvent from workup step, also gave improved yield as compared to the conventional heating, with reaction time reduced from hours to minutes. The prepared compounds were tested against A. niger and A. flavous for their antifungal activity and were found to posses good activity.

### INTRODUCTION

Organomercurials, coupled with heterocyclic compounds exhibit a wide range of pharmacological activities[1,2] like fungicidal, bactericidal etc. Organomercury compounds are known to be synthesised by diverse methodologies[3,4]. Pyrimidines are also associated with diverse biological activities[5]. Microwave assisted dry reaction technique has gained great popularity among the scientists[6,7], which can be attributed to the associated benefits of high yields, shorter reaction time and environmental compatibility without solvent usage in synthetic step. Though the workup of the reaction often requires considerable amount of organic solvents. This aspect can be overcome by the usage of water soluble inorganic salts as solid support.

In continuation of our earlier work on microwave assisted synthesis[8] of organomercurials[9] and in view of the advantages of coupling solvent free condition with MWI, it was thought worthwhile to synthesis novel barbituryl and thiobarbituryl organomercurials on $K_2CO_3$, under MWI.

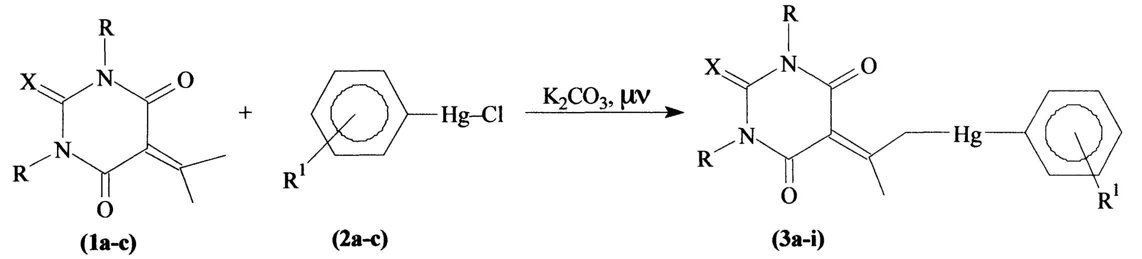

X = O ; R = H
X = S ; R = H, $C_6H_5$

$R^1$ = H, 4-$CH_3$, 4-Br

Scheme

### MATERIALS AND METHODS

Melting points were taken on an electrothermal apparatus and are uncorrected. IR spectra ($\nu_{max}$ in $cm^{-1}$) were recorded on a 1710 Perkin Elmer FTIR spectrophotometer using KBr discs. $^1H$ NMR spectra were recorded on a FT NMR Hitachi R-600 spectrometer operating at 90 MHz using TMS as internal standard (chemical shifts δ in ppm). Elemental analysis were performed on a Heraeus CHN Rapid Analyser. A Kenstar Microwave oven (Model no. OM 9925-E) operating at 2450 MHz was used. The purity of the compounds were checked on silica gel coated Al plates (Merck).

**General Procedure for the Synthesis of 5-{1-methyl-2-[aryl mercury (II)]ethylidene} barbituric/thiobarbituric acids 3 a-i**

**Method A (Microwave) :** To the equimolar (0.01 mole) solution of compound 1a-c and arylmercuric chloride 2a-c in minimum amount (about 5-10 ml) of ethanol was added 5 gm of $K_2CO_3$ and solvent was evaporated in air with occasional stirring. The dried reaction mixture was irradiated for 2-3 minutes in microwave oven (800 W, approx. temp. 90-100°C). On completion of reaction as monitored by TLC, water (25 ml) was poured into the reaction mixture with constant stirring. The product obtained was filtered, dried and recrystallized from ethanol.

**Method B (Conventional) :** The reaction mixture prepared as in method A was heated in oil bath maintained at 95-100°C for 5-6 hours. The product was worked up as in method A and was purified by fractional crystallisation from ethanol:chloroform.

### RESULTS AND DISCUSSION

Barbituric/thiobarbituric derivatives[10] 1a-c and aryl mercuric chloride[11] 2a-c have been synthesised by reported methods. The substitution of chloride by the pyrimidine derivatives 1a-c under MWI, yielded the corresponding organomercurial compounds 3a-i. The usage of $K_2CO_3$ not only simplifies the workup of the reaction as only water is required, but also eliminates the need for the external base required to neutralise the HCl evolved. Further being very poorly microwave active, $K_2CO_3$ allows larger proportion of microwave to be absorbed by the reagent as compared to other solid supports like alumina and thus leading to enhanced microwave effects contributing to much lesser reaction time (Table 1). For a comparative study, the organomercurials 3a-i were also synthesised by conventional heating using oil bath. It needed much larger time for completion and required fractional crystallisation of product to remove the uncharacterised byproducts.

**Table 1:** Reaction time and yield for compound 3 a-i

| Compound No. | X | R | $R^1$ | m.p. (°C) | Microwave Time/Yield (sec.) (%) | Conventional Time/Yield (hr) (%) |
|---|---|---|---|---|---|---|
| 3a | O | H | H | 202-203 | 150/85 | 6/60 |
| 3b | O | H | p-$CH_3$ | 170-172 | 160/90 | 6/65 |
| 3c | O | H | p-Br | 234-236 | 150/85 | 6/64 |
| 3d | S | H | H | >300 | 140/90 | 5.5/68 |
| 3e | S | H | p-$CH_3$ | 213-215 | 150/92 | 5.5/70 |
| 3f | S | H | p-Br | 250-251 | 130/90 | 5/70 |
| 3g | S | $C_6H_5$ | H | >300 | 120/92 | 5/70 |
| 3h | S | $C_6H_5$ | p-$CH_3$ | 280-282 | 135/94 | 5/80 |
| 3i | S | $C_6H_5$ | p-Br | 290-293 | 120/90 | 5/75 |

The structures of organomercurials were established on the basis of analytical and spectral data (Table II). The downfield shift of signal in $^1H$ NMR for =C-$CH_2$- from about δ 2.5 to δ 3.4 confirms the coupling of arylmercury to the pyrmidines. The elemental analysis of all compounds (Table 2) shows 1:1 stoichiometric ratio of the aryl mercury and the pyrimidine derivatives. This is the first report on the synthesis of organomercurials using $K_2CO_3$ under MWI and provides good yield in lesser reaction time with minimum usage of organic solvent.

**Table 2:** Spectroscopic data ($^1H$ NMR) for compounds 3a-i

| Compound No. | Analysis found (Calcd)% C | H | N | Hg | $^1H$ NMR ($CDCl_3$ + DMSO-$d_6$; δ, ppm) |
|---|---|---|---|---|---|
| 3a | 35.11 (35.08) | 2.72 (2.69) | 6.33 (6.29) | 45.10 (45.11) | 2.8 (3H, s, =C-$CH_3$), 3.5 (2H, s, C-$CH_2$-Hg), 7.2-7.5 (m, 5H, Ar-H) |
| 3b | 36.65 (36.63) | 3.10 (3.05) | 6.14 (6.10) | 43.73 (43.74) | 2.6 (3H, s, Ar-$CH_3$), 2.9 (3H, s, =C- $CH_3$), 3.5 (2H, s, C-$CH_2$- Hg), 7.1-7.4 (m, 4H, Ar-H) |
| 3c | 29.81 (29.79) | 2.14 (2.10) | 5.38 (5.34) | 38.33 (38.31) | 3.0 (3H, s, =C-$CH_3$), 3.8 (2H, s, C-$CH_2$- Hg), 7.6-7.9 (m, 4H, Ar-H) |
| 3d | 33.90 (33.86) | 2.62 (2.60) | 6.10 (6.07) | 43.58 (43.55) | 2.8 (3H, s, =C-$CH_3$), 3.4 (2H, s, C-$CH_2$- Hg), 7.1-7.4 (m, 5H, Ar-H) |
| 3e | 35.42 (35.34) | 2.91 (2.94) | 5.92 (5.89) | 42.3 (42.26) | 2.5 (3H, s, Ar-$CH_3$), 2.8 (3H, s, =C- $CH_3$), 3.3 (2H, s, C-$CH_2$-Hg), 7.1-7.3 (m, 4H, Ar-H) |
| 3f | 28.90 (28.91) | 2.05 (2.03) | 5.20 (5.18) | 37.20 (37.18) | 2.9 (3H, s, =C-$CH_3$), 3.6 (2H, s, C-$CH_2$- Hg), 7.2-7.5 (m, 4H, Ar-H) |
| 3g | 48.92 (48.97) | 3.30 (3.26) | 4.60 (4.57) | 32.78 (32.74) | 2.9 (3H, s, =C-$CH_3$), 3.5 (2H, s, C-$CH_2$- Hg), 7.2-7.7 (15H, m, Ar-H) |
| 3h | 49.82 (49.79) | 3.48 (3.51) | 4.50 (4.46) | 32.05 (32.01) | 2.5 (3H, s, Ar-$CH_3$), 2.9 (3H, s, =C- $CH_3$), 3.4 (2H, s, C- $CH_2$-Hg), 7.2-7.7 (14H, m, Ar-H) |
| 3i | 43.41 (43.38) | 2.71 (2.74) | 4.06 (4.04) | 29.03 (29.00) | 3.0 (3H, s, =C-$CH_3$), 3.9 (2H, s, C-$CH_2$- Hg), 7.3-7.9 (14H, m, Ar-H) |

**Antifungal Activity**

All the synthesised organomercurials 3a-i were screened for their antifungal activity against A. niger and A. flavous by paper disc diffusion method,[12] the zone of inhibition was measured in millimeters. The antifungal activity of the test compounds were compared with that of salicylic acid as standard, DMF was used as solvent.

All the organomercurial compounds displayed significant antifungal activity against both the fungi (Table 3). Thiocompounds 3d-f with phenyl substituent on pyrimidine nucleus showed better activity as compared to other derivatives. Moreover, methyl substituents 3b,e,h on the aryl mercury side chain showed good antifungal activity.

**Table 3:** Antifungal activity of organomercurials 3 a-i

| Compound No. | Asperigillus Niger | Aspergillus flavous |
|---|---|---|
| 3a | ++ | ++ |
| 3b | +++ | +++ |
| 3c | ++ | ++ |
| 3d | +++ | +++ |
| 3e | ++++ | ++++ |
| 3f | +++ | +++ |
| 3g | +++ | ++ |
| 3h | +++ | ++++ |
| 3i | ++ | ++ |
| Salicylic acid | ++++ | ++++ |

+ = 3-9 mm, ++ = 10-12 m m, +++ = 13-16 mm, ++++ = 17-21 mm.

**ACKNOWLEDGEMENTS**

The author R.V. is thankful to Council of Scientific and Industrial Research, New Delhi, for financial assistance.

**REFERENCES**

1. (a) J. Kaur, S.S. Marwaha, M.R. Boyd, G.S. Sodhi, *Neoplasma*, 1995, **42**, 191; *Chem. Abstr.*, 1996, **124**, 75530.
   (b) J. Kaur, S.S. Marwaha, G.S. Sodhi, *Rev. Latinoam Quin*, 1999, **27**(2), 65.
2. (a) F. Volna, Z. Odlerovb, M. Lacova, *Immunology*, 1973, **22**, 223.
   (b) T. Goto, H. Hayakawa, Y. Watanabe, A. Yanaji, *Eur. Pat. Appl.*, 1993, EP 572855; *Chem. Abstr.*, 1994, **120**, 164200.
3. J.J. Eisch, *Organometallic Synthesis: Non-transition metal Compounds*, Ed. J.J. Eisch & R.B. King, Vol. **2**, page 118 (Academic Press Inc., New York), 1981.
4. (a) R.C. Larock, Y.D. Lu, *Tetrahedron Lett.*, 1998, **39**, 6761.
   (b) G.B. Deacon, G.N. Stretton, *Aust. J. Chem.*, 1985, **38**, 419.
5. (a) K.S. Gulliya, U.S. *US 5,869, 494*, 1999; *Chem. Abstr.*, 1999, **130**, 163202.
   (b) L.E. Katz, W.A. Gay, U.S., *US 4,3521,806*, 1982; *Chem. Abstr.*, 1983, **98**, 215603.
   (c) F. Grams, G. Zimmermann, PCT *Int. Appl. WO 58,915*, 1998; *Chem. Abstr.*, 1999, **130**, 95559.
6. M. Kidwai, *Pure Appl. Chem.*, 2001, **73**(1), 147.
7. (a) R.S. Varma, *Green Chemistry*, 1999, 43.
   (b) A. Loupy, A. Petit, J. Hamelin, F.T. Boulet, P. Jacquault, D. Mathe, *Synthesis*, 1998, 1213.
8. (a) M. Kidwai, P. Misra, B. Dave, *Main Gp. Met. Chem.*, 2000, **23**(8), 401.
   (b) M. Kidwai, R. Venkataramanan, R.K. Garg, K.R. Bhushan, *J. Chem. Res(s)*, 2000, 586.
9. (a) M. Kidwai, P. Misra, K.R. Bhushan, *Polyhedron*, 1999, **18**, 2641.
   (b) M. Kidwai, K.R. Bhushan, *Chem. Papers*, 1999, **53**(2), 114.
10. V.K. Ahluwalia, H.R. Sharma, R. Tyagi, *Tetrahedron*, 1986, **42**(14), 4045.
11. M. Kidwai, Y. Goel, *Polyhedron*, 1996, **15**, 2819.
12. H.W. Seeley, P.J. Van Denmark, *Microbes in Action*, W.H. Freeman & Co., USA, 1972.

**Received: January 21, 2002 – Accepted: February 4, 2002 –**
**Accepted in publishable format: March 27, 2002**

